# The Intrinsic Dimension of Neural Network Ensembles

**DOI:** 10.3390/e27040440

**Published:** 2025-04-18

**Authors:** Francesco Tosti Guerra, Andrea Napoletano, Andrea Zaccaria

**Affiliations:** 1 Department of Physics, Sapienza University of Rome, 00185 Rome, Italy; 2 Istituto dei Sistemi Complessi (ISC-CNR), UOS Sapienza, 00185 Rome, Italy; andrea.napoletano1990@gmail.com (A.N.); andrea.zaccaria@cnr.it (A.Z.)

**Keywords:** intrinsic dimension, neural network, machine learning explainability, ensemble learning

## Abstract

In this work, we propose to study the collective behavior of different ensembles of neural networks. These sets define and live on complex manifolds that evolve through training. Each manifold is characterized by its intrinsic dimension, a measure of the variability of the ensemble and, as such, a measure of the impact of the different training strategies. Indeed, higher intrinsic dimension values imply higher variability among the networks and a larger parameter space coverage. Here, we quantify how much the training choices allow the exploration of the parameter space, finding that a random initialization of the parameters is a stronger source of variability than, progressively, data distortion, dropout, and batch shuffle. We then investigate the combinations of these strategies, the parameters involved, and the impact on the accuracy of the predictions, shedding light on the often-underestimated consequences of these training choices.

## 1. Introduction

Why do neural networks work? This is not an easy question to answer. During the last decades, there have been several breakthroughs in the art of crafting incrementally more efficient neural networks able to tackle problems ranging from computer vision [1,2] to automatic machine translations [3,4] and physics [5], creating different and independent fields of research. Nonetheless, even if there have been astounding advancements regarding *what* neural networks can do and regarding *how* to do it, progress about *why* neural networks can do what they do is harder to come by. Some have turned their interest to the model’s functioning to understand what the network has learned, why the model has made specific predictions, and how to interpret them [6]. In this article, we go down to a lower level to understand the role of parameters in defining the network. In this paper, we adopt the point of view of a natural scientist, and therefore, we decided to approach neural networks not as complex tools to solve problems, but as phenomena to study through experiments. So, rather than looking for novel architectures and more refined optimization strategies and settings, we take a step back and try to understand the causal connections behind the successful training of a neural network. This approach motivated our choices from the start, particularly our focus on fully connected feedforward neural networks. We aimed to remove the possible noise from complex human-biased architectures and start our investigation from the archetypal neural network that initiated this field; in this way, we had higher control over our subject of study. As a first step in our investigation, we introduced and studied the properties of the space where neural networks *live*, that is, the space explored by the possible neural networks when they are trained on the same dataset: various strategic options to train models exist, and such options generate different neural networks at convergence. We believe that the properties of the manifold generated by neural networks are connected to two open issues: the balance between over-parameterization and overfitting [7] and the representation of the loss landscape [8], which we briefly review in the following.

The loss function of a neural network is a high-dimensional, strongly non-convex function, with a dimension equal to the number of parameters used by the network. For this reason, it is not guaranteed that the training algorithm, usually the Stochastic Gradient Descent (SGD), is able to easily determine the global minimum, and, even if it does, it is not clear whether such success is attributable to the absence of local minima or to some property of the SGD [7]. Over-parameterization in neural networks, where the number of parameters (*D*) exceeds the number of training examples (*N*), allows the model to perfectly fit the training data. While this enables the network to learn the necessary patterns for prediction, it can also lead to the model fitting noise or errors present in the data, which compromise its ability to generalize [9]. Therefore, in the case of over-parameterization, a large generalization error is naturally expected. Conversely, in the opposite scenario where the training set is much larger than the number of parameters, the training algorithm forces the network to focus on the real patterns, thus avoiding overfitting. In real cases, neural networks are usually in the first of these two scenarios and yet show a great capacity for generalization [9]. Several authors have investigated this paradoxical situation. In [10], the authors show that in an over-parameterized network with ReLu activation functions, the minima of a standard mean squared error loss function are degenerate, thus making the job of the SGD easier; in [11], the authors introduce and discuss theorems on the role of over-parameterization in locating the global minimum of the loss function; in [12], the authors argue that an over-parameterization of the last layer of a neural network leads to its overfitting, while it is not detrimental to over-parameterize the previous layers; in [13], the relationship between over-parameterization and overfitting is explored through the introduction of the concept of *bias-variance trade-off* and *double descent*. Further investigations can be found in [14,15,16,17,18,19,20,21,22]. Another important field of research that can benefit from defining the space of neural networks is the analysis of the loss landscape. A graphic representation of the parameter space and the loss landscape defined therein is a valuable tool to understand the effectiveness of training algorithms and how regularization techniques impact them. In recent years, there has been a growing interest in developing precise and reliable visual representations. For example, in [23], the loss landscape of a deep convolutional neural network is analyzed in detail. In [24], the authors explore methods to generate accurate 2D and 3D visualizations of the highly multi-dimensional loss landscape, aiming to provide insights into the structure of the space and the behavior of optimization algorithms. Finally, in [8], the authors investigate the presence of multiple global minima and the convergence of the network towards different global minima given a slightly different initialization or by introducing noise in the early stages of training. They conclude that these global minima, even if corresponding to equal performance in terms of accuracy, describe different models.

We believe that a general conclusion we can deduce from this literature is that the process of training can lead to models that usually perform in a similar way and, thus, can superficially be considered similar but, on a deeper level, are very different from one another. Among the various approaches designed to enhance model accuracy and uncertainty estimation, ensemble methods play a key role. While this work focuses on analyzing the behavior of ensembles of independently trained neural networks, another widely adopted technique is Bayesian Neural Networks (BNNs). BNNs incorporate Bayesian inference to estimate a posterior distribution over network weights, providing a principled approach to uncertainty quantification. Methods such as ‘Bayes by Backprop’ [25] and dropout-based Bayesian approximation [26] offer alternative strategies to ensemble learning by treating neural network parameters probabilistically rather than relying on multiple deterministic models.

To further investigate these differences, we trained multiple instances of the same neural network archetype on the same dataset while exploring different training strategies. We considered such strategies as a source of heterogeneity among trained models and studied the space spanned by them. The first feature of this space we investigated is its intrinsic dimension, which, in our view, quantifies how much models trained for the same task actually represent different specimens of a broader population, as it measures the dimensionality of the space spanned by an ensemble of neural networks [27].

The concept of the intrinsic dimension of a set was rigorously defined in [28], and can be understood as the minimal dimension of the manifold spanned by its elements without any loss of information. In the context of neural networks, Li et al. [29] introduced one of the first operational definitions of ID, measuring the intrinsic dimension of objective landscapes by analyzing the parameter space explored during training. Their approach provides a foundational perspective on how high-dimensional loss landscapes can be effectively characterized using ID. Here, we extend this idea by focusing on ensembles of neural networks and examining how different sources of variability influence the dimensionality of the solution manifold. Several authors then came up with different strategies and algorithms to calculate it; see, for example, *TwoNN* in [30], the *Levina–Bickel* algorithm [31], *MiND_ML_* in [32], or *DANCo*, and its faster version *FastDANCo*, in [33]. These algorithms have already been applied to the study of neural networks. In [34], the authors study how the effective dimension of the internal representation of input images changes layer after layer while they are processed by a convolutional neural network. Ansuini et al. [35] follow the evolution of the intrinsic dimension in a trained network, analyzing how the inputs are transformed at every layer. Another example is [36], where the properties of training sets and neural networks are analyzed in detail from multiple points of view. Recently, Baldassi et al. [37] explored the structure of the loss function, investigating how local minima arrange to form complex structures, their generalization properties, and how algorithms based on simple gradient methods are able to find them.

In this work, we shift our perspective from trying to tinker with a neural network to understand how it works to considering a neural network as a single unitary specimen belonging to a population, i.e., an ensemble of neural networks [27] generated using different sources of variability. We study the evolution of such ensembles during training to shed some light on their evolution. Several other sources of variation can generate different models, even if they are trained on the same data. For instance, even before the training starts, the random initialization of weights already defines a manifold with a non-trivial topology and an intrinsic dimension determined by the initialization. All usual operations performed during training (dropout, batch shuffle, data distortion) modify the structure of neural networks and, as a consequence, the ensemble manifold; this is reflected in the intrinsic dimension of the set of neural networks. The role of randomness in training has been widely studied: Altarabichi et al. [38] highlight how weight initialization and structured noise influence generalization, while Zhuang et al. [39] show that even tooling choices can introduce non-trivial variability in network behavior. These factors shape the ensemble manifold and its intrinsic dimension. To systematically assess their impact, we decompose the training procedure into its fundamental steps and study how each source of variability affects the evolution of the ensemble.

## 2. Methods

In our analysis, we make use of multiple tools, which are described in the present section. The ensemble comprises fully connected feedforward neural networks, trained on the Fashion-MNIST dataset. Such networks can be represented as high-dimensional arrays defined by the learned parameters (weights and biases). The set of such arrays defines a minimal manifold whose intrinsic dimension can be computed using different algorithms. This quantity is the dimension of the ensemble of neural networks. We find that a rescaling strategy to compare and correct the different algorithms’ behavior is needed, particularly for high intrinsic dimension values. In this section, we discuss these methodological issues in detail.

### 2.1. Neural Networks

The neural networks employed in this analysis are fully connected deep neural networks that were implemented in Python 3.8, making use of the Keras library (https://keras.io/) and trained on the Fashion-MNIST dataset (https://keras.io/api/datasets/fashion_mnist/) (both accessed on 14 April 2025). They were trained to perform a 10-class classification problem and take as input a flattened 28×28 gray-scale image. For this reason, the input layer has 784 neurons, and the output layer is composed of 10 neurons. There are two hidden layers of 512 and 128 neurons, and they both use the ReLU activation function, which is a standard choice [40,41,42]). The training algorithm employed is the Stochastic Gradient Descent (SGD) with a batch size composed of 32 examples and the categorical cross entropy as a loss function as suggested in [43]. The hyper-parameters of the SGD algorithm were fixed at the Keras library default values. These neural networks were then differentiated using various variability sources, as described in Section 3.

### 2.2. Calculation of the Intrinsic Dimension

In this section, we propose a quick review of the different algorithms and strategies proposed by different authors to define and calculate the intrinsic dimension. We do not propose an original algorithm, but rather, we present to the reader the available literature in a compact and organized way to introduce the context in which we built our analysis. We believe the best starting point for this quick summary is [44], where the authors introduce a rigorous topological definition of the intrinsic dimension (ID). Given a set of N points $P_{N}$, ref. [44] considers the manifold $M\subseteq R^{d}$ that the points span, embedded in a higher dimensional space $R^{D}$ through a proper (locally) smooth map $\phi :M\to R^{D}$. This is done under the assumption that the given dataset $P_{N}$ can be expressed as $P_{N}={\left\{p_{i}\right\}}_{i=1}^{N}={\left\{\phi \left(x_{i}\right)\right\}}_{i=1}^{N}\subset R^{D}$, where $x_{i}$ are independent identically distributed (i.i.d.) points drawn from M through a smooth probability density function (pdf) $f:M\to R^{+}$. Ref. [44] defines the ID of the set of N points $P_{N}$ as the dimension *d* of the space where the manifold M is embedded.

There are numerous approaches to estimate it and provide effective numerical strategies for real-world situations that hardly fall under the rigorous boundaries of exact analytical solutions. Ref. [30] describes some of them before introducing another methodology, which we will discuss in more detail later since it is the best strategy for our needs. Let us recap the other alternatives quickly: projection techniques [45,46,47] and fractal methods [48]), both of which require a large amount of points (the scale exponentially with the ID) to give reliable estimations; see [30] for an in-depth analysis.

We exclude altogether such strategies since we have to deal with very high-dimensional embedding spaces. On the contrary, Nearest Neighbors-Based ID estimators are a class of algorithms that are more suitable for this task since they require fewer points to work properly. In particular, they are based on the assumption that given a ball of radius *r* centered in a point *x* of a manifold M, $B_{d}\left(x,r\right)$, for an *r* small enough, points uniformly extracted from $B_{d}\left(x,r\right)$ approximate well enough the local structure of M; see [44] for the details.

We present some of the most common algorithms here, briefly discussing their assumptions and their points of strength, and then apply them to our use case and compare their performance. In the following, we will consider ensembles $P_{N}$ of *N* neural networks, each one defined by its own *D*-dimensional array $p_{i}$, where *D* is the number of parameters of the neural networks, and d is the unknown intrinsic dimension of this ensemble.

#### 2.2.1. TwoNN

The TwoNN algorithm, proposed by Facco et al. [30], is based on the minimal hypothesis that only the distances of the two closest neighbors from any given point matter to estimate the intrinsic dimension of a set. The strength of this hypothesis is to make the estimation of the ID less sensible to the ensemble’s inhomogeneities, anisotropies, and irregularities. Let us consider an item $p_{i}$, i=1,…,N, of an ensemble $P_{N}$ of which we want to calculate the ID; let $r_{i}^{\left(1\right)}$ and $r_{i}^{\left(2\right)}$ be the Euclidean distances between the item $p_{i}$ and its first and second neighbors, respectively. Then, the ratio $\mu_{i}=r_{i}^{\left(2\right)}/r_{i}^{\left(1\right)}$, with *N* numbers of items in the set, follows a Pareto distribution:

(1) $$f\left(\mu_{i}\right)=d\mu_{i}^{-d-1}1_{\left[1,+\infty \right]}\left(\mu \right),$$

where d is the dimension of the manifold spanned by the items of the set and 1 is the characteristic function.

The TwoNN estimator treats the ratios $\mu_{i}$’s as independent, i=1,…,N, and estimates the overall ID d on the entire dataset, employing a least-squared approach. Ref. [30] proposes to consider the cumulative distribution of each $\mu_{i}$, obtained by integrating Equation (1), given by $F\left(\mu_{i}\right)=\left(1-\mu_{i}^{-d}\right)1_{\left[1,+\infty \right]}\left(\mu_{i}\right)$, and to linearize it into $\log\left(1-F\left(\mu_{i}\right)\right)=-d\log\left(\mu_{i}\right)$. Then, a linear regression with no intercept is fitted to the pairs ${\left\{-\log\left(1-\tilde{F}\left(\mu_{i}\right)\right),\log\left(\mu_{i}\right)\right\}}_{i=1}^{N}$, where $\tilde{F}$ denotes the empirical cumulative distribution of the sample μ sorted by increasing order. To enhance the estimation, the authors also suggested discarding the last percentiles of the ratios $\mu_{i}$’s, usually generated by observations that fail to comply with the local homogeneity assumption. The requirement of local uniformity only in the range of the second neighbor is an advantage with respect to competing approaches where local uniformity is required at larger distances. In datasets characterized by sharp boundaries, such boundaries introduce a critical violation to the assumption of local uniformity. Consequently, the estimates are affected [30].

#### 2.2.2. MLE

The MLE algorithm, one of the most cited estimators, proposed by Levina and Bikel [31], treats the neighbors of each point $p_{i}\in P_{N}$ as events in a Poisson process and the Euclidean distance $r^{\left(j\right)}\left(p_{i}\right)$ between the query point $p_{i}$ and its *j*th nearest neighbor as the event’s arrival time. Since this process depends on d, MLE estimates the ID by maximizing the log-likelihood of the observed process [44]. In practice, a local ID estimate is computed as

(2) $$d\left(p_{i},k\right)={\left[\frac{1}{k-1}\sum_{j=1}^{k-1}\log\frac{r_{k}\left(p_{i}\right)}{r_{j}\left(a\right)}\right]}^{-1}.$$

Assuming that each item of the set belongs to the same manifold, the global ID can be written as:

(3) $$d\left(k\right)=\frac{1}{N}\sum_{i=1}^{N}d\left(p_{i},k\right),$$

where *N* is the number of items in the set. To remove the dependency from the parameter *k*, the authors suggest averaging over a range of values for the number $k=k_{1},\ldots ,k_{2}$ of first neighbors to obtain a more robust final estimate of the ID:

(4) $$d=\frac{1}{k_{2}-k_{1}+1}\sum_{k=k_{1}}^{k_{2}}d_{k}.$$

MLE underestimates the ID for high ID values. This problem is shared by all dimensional estimators. Ref. [31] argues that one reason is that the MLE approximation is based on the assumption that enough points fall into a small sphere, but, to be true, the higher the ID, the larger the sample points taken should be. In some cases, there are also boundary effects to take into account, which are more severe for higher dimensions.

#### 2.2.3. MiND_ML_

The MiND_ML_ algorithm, proposed by Lombardi et al. [32], exploits the pdf g(r;k,d) describing the distance $r^{\left(1\right)}\left(x\right)$ between the center *x* of a ball $B_{d}\left(x,r\right)$, x∈M, $r\to 0^{+}$ and its nearest neighbor, where g(r;k,d) as function of d can be shown to be $g\left(r;k,d\right)=kdr^{d-1}{\left(1-r^{d}\right)}^{k-1}$. A maximum likelihood approach computes the ID estimator. Underestimation of the ID is still present for high ID values, i.e., an ID ≥10 like in the MLE algorithm. Both [31,32] agree on a qualitative explanation of such bias: ID estimators based on nearest-neighbor distances are often founded on the hypothesis that the available data are unlimited, which is never the case in practical applications.

#### 2.2.4. DANCo

Trying to overcome the drawbacks of MiND_ML_, Ceruti et al. [33], building on the work of [32], propose the DANCo algorithm, which reduces the underestimation effect by combining an estimator employing normalized nearest-neighbor distances with one employing mutual angles. To reduce the bias between the analytical pdf *g* and the estimated one $\hat{g}$, DANCo compares the statistics estimated on $P_{N}$ with those estimated on (uniformly drawn) synthetic datasets of known ID. The comparisons are performed by two Kullback–Leibler divergences applied to the distribution of normalized nearest neighbor distances g(r;k,d), where $g\left(r;k,d\right)=kdr^{d-1}{\left(1-r^{d}\right)}^{k-1}$, and the distribution of pairwise angles q(x;ντ), q(x;ντ) is the von Mises–Fisher distribution [49] with parameters ν,τ. Hence, the estimated ID $\hat{d}$ is the one minimizing the sum of the two divergences:

(5) $$\hat{d}=\underset{1\leq d\leq D}{arg min}\mathrm{KL}\left(g_{P_{N}},g_{synt}\right)+\mathrm{KL}\left(q_{P_{N}},q_{synt}\right)$$

The computation of the first *k* neighbors for each point of the synthetic d-dimensional dataset for d=1,…,D entails, especially for large values of *D*, high computational time. For this reason, Ceruti et al. [33] proposed a “fast” version of DANCo: FastDANCo. The acceleration is given by precomputing the variables that do not depend on the dataset, but only on *N* and d: $d_{{MiND}_{\mathrm{ML}}}^{synt},\nu_{d}^{synt},\tau_{d}^{synt}$. Hence, given *k*, the variables $d_{{MiND}_{\mathrm{ML}}}^{synt},\nu_{d}^{synt},\tau_{d}^{synt}$ are calculated for various values of d and *N* and the dependence of each variable on d and *N* is described using suitable fitting functions. Due to FastDANCo’s significant computational time advantage over DANCo and substantially equal accuracy, we will employ the “Fast” version compared to the other algorithms.

### 2.3. Rescaling the Algorithms

**Figure 1:**
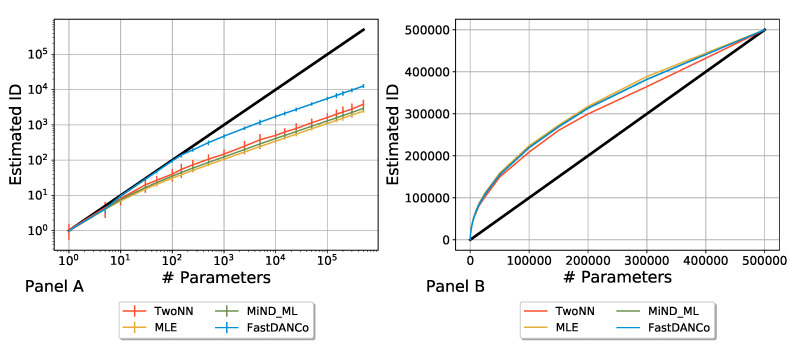
Each algorithm underestimates the ID for larger values of the ID, Panel (**A**). To compare results between algorithms and to apply them to neural networks, which have a large number of parameters, we imposed a rescaling of the output of each algorithm, forcing them to be equal to 500,000, which is the largest number of parameters employed, when the ID is exactly 500,000, Panel (**B**).

Before computing the ID of the ensembles, we tested the performance of the algorithms presented in the previous section and conducted a systematic comparison of the results they yielded. This was particularly important since such algorithms are usually applied in situations in which both the embedding and the intrinsic dimensions are much smaller than in our case. We built a synthetic dataset that gives us full control over the ID: a set of N=100 vectors with a variable embedding dimension. Each element was randomly generated with a uniform distribution, thus ensuring that the intrinsic dimension was equal to the embedding dimension by construction. In other words, by changing the number of elements of each vector, we can simulate different numbers of independent parameters to study the behavior of the algorithms for various values of the ID. We let the simulated embedding dimension range between 1 and 500,000 and repeated this analysis multiple times to estimate the mean and standard deviation of the calculated ID for every algorithm. The results are shown in Figure 1, the left panel. All the algorithms revealed a consistent underestimation of the intrinsic dimension. For this reason, we introduced a rescaling of the result of each algorithm, forcing the calculated ID to be equal to the actual ID when the real ID was equal to 500,000; see Figure 1, the right panel. The general rescaling transformation is represented in the following equation:

(6) $${\mathrm{ID}}_{R}^{a}\left(n\right)=n_{max}\frac{{\mathrm{ID}}^{a}\left(n\right)}{{\mathrm{ID}}^{a}\left(n_{max}\right)}$$

where ${\mathrm{ID}}_{R}^{a}$ is the rescaled estimated intrinsic dimension, *n* is the number of parameters that can range between 1 and $n_{max}$ = 500,000, ${\mathrm{ID}}^{a}$ is the intrinsic dimension estimated by the algorithm *a* before rescaling, and the label *a* indicates a specific algorithm.

For a greater number of vectors, the performance of all the algorithms used to calculate the intrinsic dimension increased: they were less prone to underestimate the ID and became more reliable; however, we chose N=100 to reflect the number of neural networks composing each ensemble, since training a larger number of models would be too computationally demanding. We point out that the rescaling procedure used in Figure 1 Panel B is not used in the rest of the paper, as it is solely adopted here to show the coherence between the different estimation approaches.

In the following analysis, we will focus on the FastDANCo algorithm, as it is the most reliable at higher dimensions, as shown in Figure 1 Panel A; moreover, it offers the best trade-off between accuracy and computational complexity.

## 3. Results

During the training of a neural network, many options and techniques are available to speed up convergence, reduce overfitting while increasing generalization, and optimize the search for a stable and reliable minimum in the loss landscape. The specific choice is left to the practitioner, their expertise, and the often a posteriori assessments in the literature. A different choice will result, at the end of the training, in a different model (that is, a different set of learned parameters). In this section, we focus our attention on the most common strategies and settings employed during a standard training procedure that result in different convergence models: the random initialization of weights, batch shuffle and batch size tuning, and dropout rate setting. All these sources of variability will produce different neural networks. The questions we want to answer are as follows: what is the impact of each one of said operations on the training? How do they compare to each other? What happens when we combine them? The intrinsic dimension of the manifold, spanned by an ensemble of neural networks trained with different sources of variability, offers us an objective instrument to shed some light on these questions. Indeed, the ID quantifies the size of the configuration space each method opens up: the larger the ID, the more the ensemble’s neural networks differ.

All possible training strategies were evaluated on ensembles of 100 neural networks with the architecture described in Section 2.1. All 100 networks of the ensemble were trained using one technique, or a set of techniques, at a time, while during training, we estimated the evolution of the ID. For instance, when we adopted dropout to train our networks, all the other sources of variability were fixed: all the networks were initialized with the same parameters, and batch shuffling and the different sources of variation were turned off. The ensemble of networks can be seen as a collection of 100 vectors and calculated the ID making use of the FastDANCo algorithm since the embedding dimension was huge. We checked that the main results do not change using different algorithms; see Appendix A for more details. To express a neural network as a vector, we followed a simple rule: at each epoch (an epoch being the number of training steps required to see the whole dataset, thus depending on the batch size) of the training, we went through the network layer by layer, we converted the weight matrix into a vector by flattening it, we concatenated the bias vector, and we moved to the next layer repeating the operation. In the end, for each neural network we obtained one vector of D=468,874 elements. So, the neural networks were defined in a space equal to the embedding dimension D; we aimed to calculate the ID of the manifold they spanned, which, naturally, evolved during the training.

### 3.1. Computation of the ID Induced by Different Variability Sources

We aimed to understand the effect of the single strategies of learning, each of which can be seen as a variability source. So, we generated one ensemble of neural networks activating only one variability source at a time: random initialization, batch shuffling, dropout, or random data distortion. Unless otherwise specified, all models were initialized with the same set of parameters (thus spanning a manifold of intrinsic dimension 0). Furthermore, they were trained with the original (not modified) images, with a fixed batch size of 32 images and in a fixed batch order. Lastly, the dropout rate was set to zero. In this way, we could test how the ID increased and evolved during training by turning on a particular strategy at a time. In particular, we tested the effect on the ID of the following variability sources:

- **Random parameter initialization**. Each network was initialized with random parameters. Note that in this case, the starting (i.e., epoch zero) ID was the highest possible, and it was equal to the embedding dimension D. We refer to this ensemble as “Rand. Init.”;
- **Random shuffling of the batches during the training**. The order in which the SGD saw batches of the training set was random for each network. We refer to this ensemble as “Batch Shuffle”;
- **Random exclusion of neurons during the training**. We applied a dropout with a dropout rate of 0.5. We refer to this set as “Dropout”;
- **Random distortion of the training set images**. The images of the training set were randomly distorted, so each model was trained on slightly different data. We refer to this ensemble as “Distorted”, as was done by Ciregan et al. in [50]. See Appendix B for more details.

**Figure 2:**
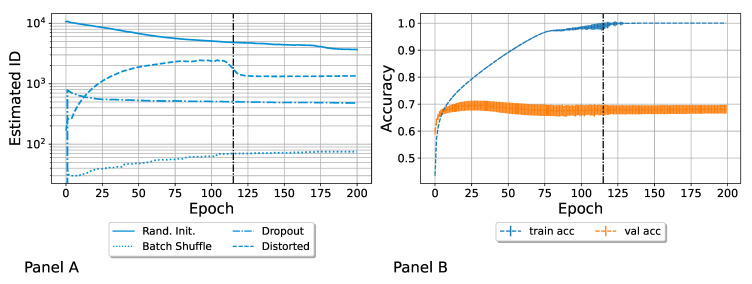
Intrinsic dimension (ID) as a function of the training epoch for different ensembles, generated by as many variability sources (Panel (**A**)). The induced heterogeneity spans different orders of magnitude. The distortion of the training data can produce a higher ID than the dropout. We stress a drop in the “Distorted” ensemble with a dark vertical line. In Panel (**B**), we report the accuracy (with error bars) as a function of the epoch for the “Distorted” ensemble and the vertical line indicates the same epoch, in which the accuracy also tends to 1. This suggests that the ID drop could be related to a transition to an overfitting regime.

**Figure 3:**
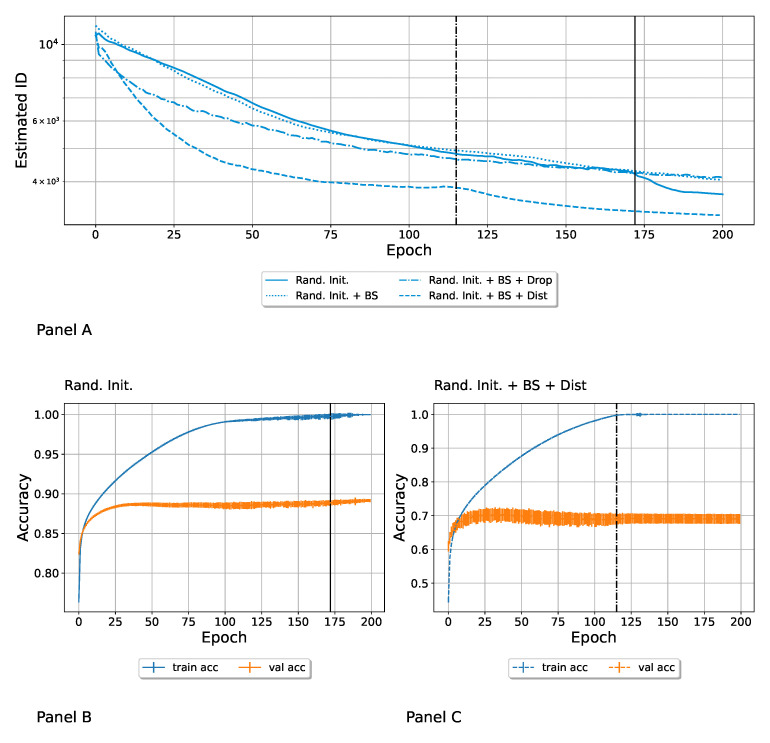
Panel (**A**): Intrinsic dimension (ID) as a function of the epoch for different ensembles generated by random initialization plus other variability sources. Batch shuffle had a minimal effect. The dropout regularizes the training so that the networks were closer to the minimum and were more similar despite the added variation. The networks trained on the distorted datasets quickly reached overfitting, so they converged faster to a similar configuration that reproduced the training data structure, thus quickly decreasing the ID. Panel (**B**): the drop in the “Rand. Init.” ensemble occurred at the same epoch in which the accuracy plot shows a transition to an overfitting region (marked by the continuous vertical line). Panel (**C**): also in the case in which we added the batch shuffle and the distortion to the source of variability coming from the random initialization, the drop in the ID occurred at the same epoch of the transition to the overfitting region (marked by the dashed line).

Each variability source generated an ensemble. Each neural network was trained for 200 epochs, and the ID of the ensemble was calculated at each epoch. The resulting evolution is depicted in Figure 2, Panel A, where each line corresponds to a variability source, and the corresponding ID as a function of the epoch is reported. The first point to notice is that each ensemble had a characteristic order of magnitude for its intrinsic dimension; the random initialization led to a higher ID, followed by training data distortion and dropout. The random shuffling of the batches during the SGD was the only strategy that led to a monotonic increase in the ID, even if the induced heterogeneity was very low. The random initialization, as expected, immediately generated a very heterogeneous ensemble, which spanned a high dimensional manifold, whose ID was already decreased by a factor of ∼15 in the first epoch. This decrease continued because of the training; however, the ID was always higher than the one relative to the other ensembles. On the contrary, we found a steep growth of the ID from epoch 0 to epoch 1 for the “Distorted” and the “Dropout” configurations, which started from identical parameters (which led to ID = 0). In the case of “Dropout”, the first epoch was sufficient for the ID to reach its characteristic order of magnitude. In Appendix C, we report a focus of the first epoch, where we followed the evolution of the ID batch by batch. In the case of randomly distorted trained images, the ID first increased and then it started to decrease. We marked the epoch when this drop happened with a vertical dark line. In Panel B, we show that this happened right before the average accuracy of the distorted ensemble in the training converged to 1. This drop also appeared in the Rand. Init.; the scale of Figure 2 makes this difficult to notice, so please refer to Figure 3, where this behavior is more apparent. Our tentative explanation is that the drop in ID marks a transition to an overfitting regime. In Figure 3, we focus on the random initialization scenario and also turned on the other variability sources to evaluate the impact of applying both. This particular order of adding variation sources is the reason for the popularity of the techniques adopted. Randomly initializing network parameters and randomly resampling dataset subsets are perhaps the most commonly used methods to create model variation in members of neural network ensembles [51]. In particular, we added the following ensembles to the “Rand. Init.” case:

- **Random initialization + Batch Shuffle**. Each network was initialized with random parameters, and the order in which batches were presented was randomized for each network differently. We refer to this ensemble in the figure as “Rand. Init. + BS”;
- **Random initialization + Batch Shuffle + Dropout**. Each network was initialized with random parameters, the batch order was random, and the dropout rate equal was 0.5. We refer to this ensemble as “Rand. Init. + BS + Drop.”;
- **Random initialization + Batch Shuffle + Distortion**. Each network was initialized with random parameters, the batch order was random, and the images were randomly distorted (for each network in a different way). We refer to this ensemble as “Rand. Init. + BS + Dist.”;

In Panel A of Figure 3, we compare the evolution of the IDs at different epochs. Adding other techniques accelerated training and converged more quickly to the solution. The intrinsic dimension decreased since the networks were more similar in the minima. Note that all four of these configurations started from the highest possible value of the ID by construction, and decreased during learning. Among all the strategies proposed, presenting randomly distorted images during training had the biggest impact in reducing the ID of the manifold spanned by the neural networks, thus making them more similar to each other faster. Moreover, we found a steep drop in the ID, noticeable in the “Rand. Init.” scenario. In Panel B of the figure, we show that, again, this happened right before the accuracy on the training set reached 1, thus reinforcing the hypothesis that such a steep drop in the ID marked the passage into an overfitting regime where the networks started learning random noise in the training set rather than a meaningful signal. In Panel C, we can observe that the “Rand. Init. + BS + Dist.” ensemble experienced the same regime change around epoch 115, i.e., a transition to an overfitting region. In both panels B and C, we show the error bars corresponding to the standard deviation over the network ensemble.

We note that even if the distortion of images usually improved the generalization capacity of the models, in our case, we wanted to show a specific effect on the ID of the ensemble; consequently, we used a relatively high distortion factor. This allowed us to clearly show the effect of this factor on the ensemble ID estimation but caused a drop in the prediction accuracy.

**Figure 4:**
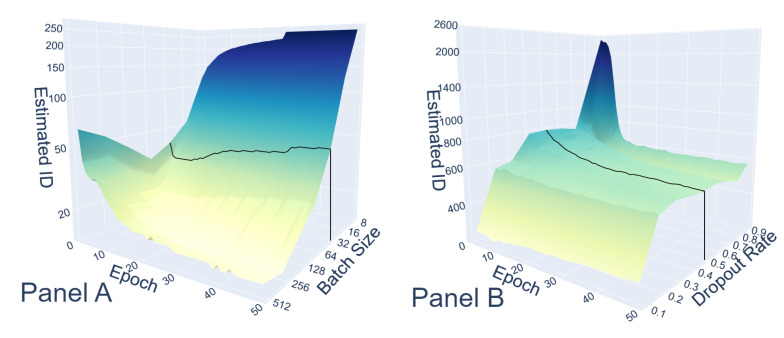
Trend of the estimated ID in the “Batch Shuffle” case as the batch size changed. The smaller the size, the greater the amount of shuffling, hence the ID and Panel (**A**). The peak is justified by the high dropout rate, making the few activated neurons much more different in each network instance. The regularization effect of the dropout is highlighted by the smooth decrease for every dropout rate, Panel (**B**). In both panels, a solid black line has been added to aid perspective viewing and refer to the respective trends reported in Figure 2.

Thereafter, we wanted to evaluate the relative intensity of the possible sources of variability and the dependence from the specific value of the involved parameters. This allowed for a visual representation of the function linking the ID to the various parameters. In Figure 4, we shift our attention on the impact of different values of the batch size with random batches and no dropout, Panel A, and other values of the dropout rate with fixed batches of 32 images, Panel B, on the properties of the networks’ manifold. In both cases, the only sources of variation turned on were those characterizing the focus of the study: batch shuffle and dropout, respectively. Let us discuss the results: on the one hand, a smaller batch size implies longer epochs; longer epochs imply a more significant number of random batches, thus allowing networks to diverge more from each other and span a manifold with a greater ID that keeps increasing epoch after epoch. On the other hand, a high dropout rate implies that, at each time step of every epoch, there is a large number of randomly deactivated neurons, so it is natural to expect high diversity between individual networks for higher values of the dropout rate. The peak that we see in Panel B at the beginning of the training is due exactly to this effect; at each step, only 10% of neurons were active, thus adding extreme variety in the network manifold. As the training went on, the regularizing effect of the dropout helped the networks converge to optimal solutions, and the ID decreased. It is interesting to notice that as the dropout ranged from 0.1 to 0.9, at the beginning of the training, the ID did not increase monotonously but displayed a local minimum between the dropout rate of 0.7 and 0.8, probably because such values offered the best regularization effect right from the beginning.

### 3.2. Accuracy of Heterogeneous Ensembles

**Figure 5:**
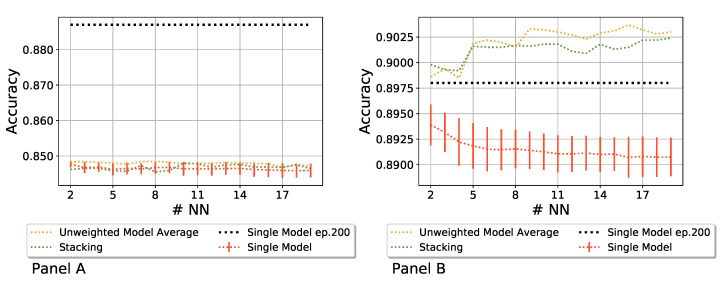
Panel (**A**): a low-dimensional ensemble is outperformed by a single network. Panel (**B**): A high-dimensional ensemble outperforms the single networks. To show a fair comparison in terms of computational effort, the single model was trained for 200 epochs, while the models in the ensemble were trained for 50 epochs. Two ensembling techniques were implemented: Unweighted Model Average and Stacking. These sets were made with an increasing number of networks (#NN). In Panel (**A**), we show the results of the “Batch Shuffle” ensemble with batch size 512, while in Panel (**B**), we show the “Batch Shuffle” ensemble with batch size 8.

After exploring different strategies to train neural networks and disentangle their effect as variability sources on the evolution of the networks’ manifold, we wrapped up the analysis by studying the relationship between the ID and the possible benefits of making predictions with an ensemble of neural networks. To provide a fair comparison between ensembles and single neural networks, we trained the latter for more epochs to simulate two scenarios with roughly similar computational effort. Moreover, we compared two ensembles with different IDs by changing the batch size, following the results obtained in the previous section. We trained an ensemble formed by a variable number of networks for 50 epochs and averaged their predictions through the *Unweighted Model Average* [52,53] and the *Stacking* [54,55] techniques, and compared the performance with a single network trained for 200 epochs and with the average performance of the single networks forming the ensemble, so *without* combining their predictions into a unique consensus score. In particular, we tested two configurations, whose results are shown in Figure 5: the “Batch Shuffle” ensemble with a batch size of 512 (panel A, ID = 15) and the “Batch Shuffle” ensemble with a batch size of 8 (panel B, ID = 251), both trained for 50 epochs. These figures show the prediction accuracy as a function of the number of neural networks in the ensemble. In panel A, we show that the low-dimensional ensemble performance was always lower than the single network; in panel B, the single models in the ensemble had, on average, a better performance than in the previous scenario and, more importantly, combining them in a high-dimensional scenario consistently outperformed the benchmark model trained for 200 epochs, even if only by a small amount. We can conclude that similar networks form ensembles with a small ID, and combining them does not improve the overall performance because there is insufficient heterogeneity. On the contrary, a higher ID denotes more variety among networks, each with different strengths; thus, combining them, even if trained for a shorter time than traditional models, can offer a better prediction performance. However, even if a high ID suggests that the ensemble will likely perform better than a single network, it is not a sufficient condition for improved performances. While a high ID allows for a broader exploration of the solutions space, each network still needs to capture specific, complementary aspects of the database so that by combining them, one obtains an improved prediction.

In real application scenarios, where regularization techniques are combined meaningfully and not isolated, this test shows how one can use the ID to keep track of the evolution of the ensemble and possibly define new training strategies. A sketch of a possible ensemble training procedure could be as follows:

1. Identify the best architecture for a given problem;
2. Build a suitable ensemble of networks for the analysis;
3. Keep track of the evolution of the ID of the ensemble to fine-tune the training strategy to explore a large portion of the solution manifold and identify the optimal ID for the specific problem;
4. Average the prediction of the network ensemble and compare its accuracy to a benchmark, for example, a single model, and see if the ensemble forecasting outperforms single model forecasting.

We will explore such strategies in future works.

### 3.3. Comparison with Hidden Representation

After analyzing the intrinsic dimension (ID) of neural networks as atomic entities, we extended our investigation to examine the ID of the data processed by individual hidden layers, as previously reported in [35]. In this way, we could connect our analysis to the established literature and determine whether the patterns previously observed at the network level also appeared at the layer level.

We calculated the intrinsic dimension of hidden layer representations across four different ensembles using 20 images from Fashion MNIST. We examined the representations in a hidden layer across all 100 networks in each ensemble for each image and computed the ID. The results were then averaged across all 20 images for each ensemble.

**Figure 6:**
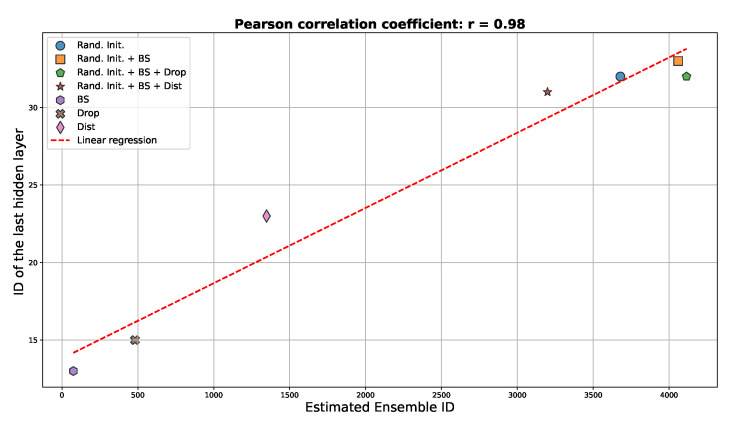
Correlation between data hidden representation ID and full network ID across different ensembles. The scatter plot demonstrates the strong relationship (correlation coefficient = 0.98) between hidden layer ID values after 200 training epochs and the corresponding full network ID.

In Figure 6, we show that the data hidden representation ID followed the same pattern observed for the complete networks, with a strong correlation coefficient of 0.98.

These results align with the ordering of curves presented in Figure 2A, confirming that the factors affecting the intrinsic dimensionality at the network level similarly influenced individual hidden layers [35].

## 4. Discussion

Training neural networks requires an educated balance between science and craftsmanship. On the one hand, multiple general training strategies and complex architectures that can be adapted to specific contexts have been developed; on the other hand, to obtain optimal results, a precise tuning strategy—strongly dependent on the problem at hand and the dataset available—is often required. This behavior contributes to the general conception that neural networks are potent yet mysterious black boxes that are difficult to interpret and explain. In the global effort to understand why neural networks work, some studies focus on the transformation that they induce on the training data layer by layer up to the output layer, and the dimension of the dataset is studied as it is processed. Other studies analyze the activation of specific areas of a trained network to produce a mapping between clusters of neurons and dataset properties. The common denominator of such studies is that neural networks are usually taken apart to study individual components’ behavior or single layers’ behavior. We wanted to contribute to this field by considering a neural network as an individual atomic element and studying their collective behavior. Trained neural networks live in a manifold embedded in the space of parameters suited, on the one hand, to reproduce the manifold of the training data, and, on the other, to generalize to new data without overfitting. All training strategies concur to project neural networks to an optimal manifold of the space of parameters that allows for generalization and grants the ability to make predictions and forecasts. Identifying and defining this optimal manifold is quite a daunting goal, but we can assume that a trained network that can successfully perform its tasks is close to it. When training an ensemble of networks, each specimen will converge to the optimal manifold and form a limited representation. We have shown how, through the intrinsic dimension, it is possible to obtain an idea of the size of the portion of the optimal manifold made available to the network ensemble by the training.

We note that several important aspects of the solution manifold remain to be explored in future work. One key question is whether the obtained manifold is locally or globally rugged, or instead forms a smoother structure where solutions are easily connected. Studies on loss landscapes suggest that different minima found by neural networks are often connected by low-loss paths, enabling efficient ensembling and reducing barriers between solutions [56]. Similarly, evidence from energy landscape analyses indicates that minima are not isolated but instead lie on relatively smooth, connected surfaces [57]. Investigating whether neural network ensembles exhibit similar connectivity, or whether training variability leads to more fragmented solution spaces, would provide valuable insights. Additionally, we plan to investigate how small perturbations affect model performance and solution stability. This analysis will quantify how far perturbations move solutions from the optimal manifold and test our hypothesis that models with lower intrinsic dimensionality are more susceptible to perturbations. Finally, examining the norm of the weights would provide insights into how the loss landscape is explored during training. By tracking how weight norms evolve and comparing across different initialization strategies, we can potentially explain why specific initializations perform better than others. These considerations remain speculative at this stage and will be empirically verified in future research to deepen our understanding of neural network solution spaces.

## Figures and Tables

**Figure A1:**
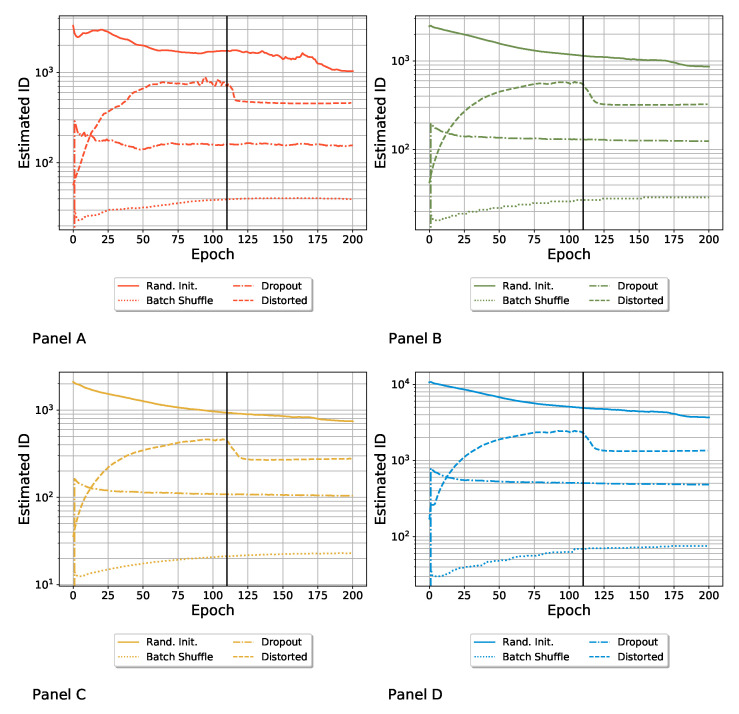
Robustness test with respect to a change in the algorithm to compute the ID. We repeat the exercise reported in Figure 2 but using TwoNN (Panel (**A**)), MiND_ML_ (Panel (**B**)), MLE (Panel (**C**)), and FastDANCo’s (Panel (**D**)). All the algorithms showed the same qualitative behavior with just a scaling factor of difference. Notably, all algorithms show a consistent drop in the ID for the distorted ensemble around epoch 110 (indicated by the black vertical lines).

**Figure A2:**
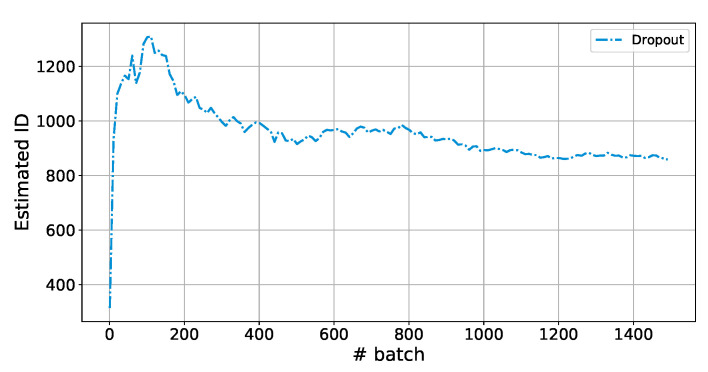
Zoom into the first epoch of “Dropout” to investigate the sudden increase in the ID found in Figure 2. At the very beginning, after the evaluation of a few hundred batches, the dropout made the neural networks of the ensemble different. After that, the regularizing behavior of the dropout emerged, causing the decrease in the ID.

**Figure A3:**
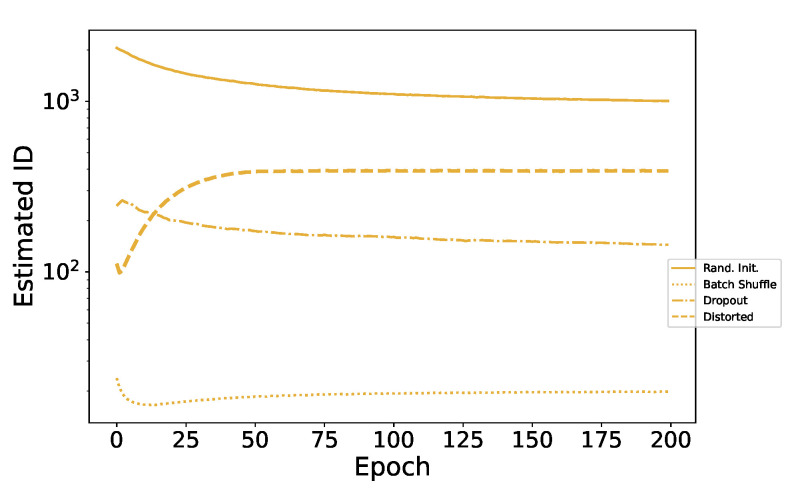
Intrinsic dimension (ID) as a function of the training epoch for different ensembles, generated as explained in Figure 2 with the MNIST dataset. No significant differences exist with the more complex dataset explored in the paper.

**Figure A4:**
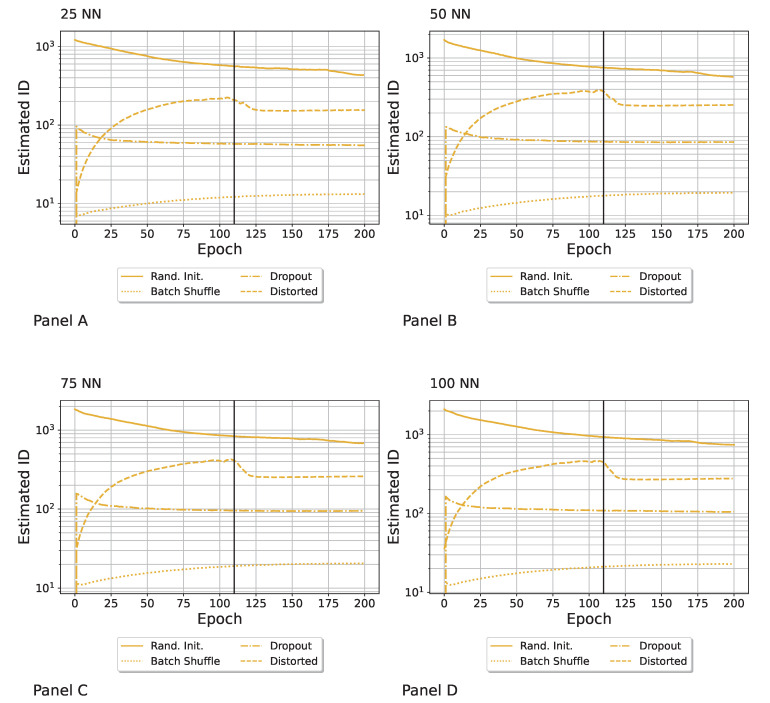
Robustness with respect to ensemble size. We show the results for ensembles composed of 25 (Panel (**A**)), 50 (Panel (**B**)), 75 (Panel (**C**)), and 100 (Panel (**D**)) neural networks. The qualitative trends observed in the main text remain consistent across different ensemble sizes, with coherent quantitative changes. The vertical lines indicating the drop in ID for the distorted ensemble around epoch 110 are also present here.

## Data Availability

The code used in this study is available in the following GitHub v1.0.0 repository: https://github.com/tgfrancesco/NN_intrinsic_dimension (accessed on 14 April 2025) [58].

## Abbreviations

The following abbreviations are used in this manuscript:

ID	Intrinsic Dimension
NN	Neural Network
SGD	Stochastic Gradient Descent
Rand. Init.	Random Initialization
BS	Batch Shuffle
Drop.	Dropout
Dist.	Distortion

## Appendix A. Comparing Algorithms

We rely on this appendix to discuss a couple of minor points left out of the main paper as not to break the flow of reasoning. These include a comparison between different algorithms to calculate the intrinsic dimension, further details on the image distortion technique considered in our analysis, and a focus on the first epoch of training to explain the behavior of the intrinsic dimension.

As discussed in Section 2.3, rescaling the ID estimations provided by different produced a good collapse of the respective functions. In the main text, we only report the results relative to the FastDANCo algorithms, which provides a good trade-off between accuracy and computational time when the ID of the manifold is far larger than the number of elements of the set. In Figure A1, we report the results for the remaining algorithms reproducing the test reported in Figure 2. Even if different scales of dimensionality were present, all the algorithms captured the same qualitative behavior. In particular, all algorithms presented the same rankings of the IDs and the same drop at epoch ∼110 for the distorted ensemble, thus proving that it is not a byproduct of the algorithm choice. The noisy trends of TwoNN were due to its extreme sensitivity to measurement noise since it only involves a narrow neighborhood of each point [59]. We can conclude that our findings are robust with respect to the choice of different algorithms to compute the ID.

## Appendix B. Dataset Distortion

To train the neural networks of the “Distorted” ensemble and of the “Rand. Init. + BS + Dist.” one, we randomly distorted the images of the training set. Hence, each network was trained on a different set. We used the ImageDataGenerator class of Keras to perform rotations, shifts along the horizontal and/or vertical axis, zooming, and shear transformations. In particular, the rotation used was up to ±5°, similar to the one used by Ciresan et al. in [50] to study the CIFAR10 dataset, the translation was up to ±25%, the zoom was in the range [1,1.5], and, to create the perception angles, we chose a shear intensity of 45°.

## Appendix C. Zoom in the First Epoch

The strong discontinuity found in the trend of the estimated intrinsic size of the “Dropout” ensemble (Figure 2) can be explained by considering only the first epoch of the training and studying the ID of the ensemble every 10 batches. We found an explosive ID growth at the beginning of the training and a subsequent slow decrease that continued in the following epochs. The quick increase in the ID, because a random half of the neurons were deactivated at each step, explains why there was already a high value of the ID at the end of the first epoch.

## Appendix D. MNIST

To prove the robustness of our conclusions, we repeated the computation of the intrinsic dimension for the MNIST dataset. In Figure A3, we show the results, particularly the intrinsic dimension as a function of the training epoch. The observed behavior is very similar to the one observed in Figure 2.

## Appendix E. Numerical Comparison of Loss and Accuracy

Table A1 and Table A2 show the numerical values of the loss and accuracy at the end of training with the corresponding standard deviation.

**Table A1:** Train and validation loss at epoch 200 with the error calculated over 100 networks.

	Train Loss	Val Loss
Rand. Init	0.004 ± 0.001	0.644 ± 0.018
Rand. Init. + BS	0.003 ± 0.001	0.553 ± 0.009
Rand. Init. + BS + Drop	0.382 ± 0.002	0.321 ± 0.002
Rand. Init. + BS + Dist	0.003 ± 0.0001	2.318 ± 0.157

**Table A2:** Train and validation accuracy at epoch 200 with the error calculated over 100 networks.

	Train Acc	Val Acc
Rand. Init	1.0000 ± 0.0002	0.892 ± 0.002
Rand. Init. + BS	1.0000 ± 0.0003	0.897 ± 0.001
Rand. Init. + BS + Drop	0.858 ± 0.001	0.882 ± 0.001
Rand. Init. + BS + Dist	1.0000 ± 0	0.690 ± 0.014

## Appendix F. Robustness with Respect to the Number of Neural Networks

In the main text of the manuscript, we investigated ensembles with a fixed number of 100 neural networks. To investigate the robustness of our conclusions with respect to the number of neural networks included in each ensemble, we trained new ensembles with a variable number of elements. The results are shown in Figure A4. Clearly, the qualitative behavior was stable with only highly coherent quantitative changes.

## References

[1] Voulodimos A., Doulamis N., Doulamis A., Protopapadakis E. (2018). Deep learning for computer vision: A brief review. Comput. Intell. Neurosci..

[2] Khan S., Naseer M., Hayat M., Zamir S.W., Khan F.S., Shah M. (2021). Transformers in vision: A survey. ACM Computing Surveys (CSUR).

[3] Bahdanau D., Cho K., Bengio Y. (2014). Neural machine translation by jointly learning to align and translate. arXiv.

[4] Stahlberg F. (2020). Neural machine translation: A review. J. Artif. Intell. Res..

[5] Guidarelli Mattioli F., Sciortino F., Russo J. (2023). A neural network potential with self-trained atomic fingerprints: A test with the mW water potential. J. Chem. Phys..

[6] Montavon G., Samek W., Müller K.R. (2018). Methods for interpreting and understanding deep neural networks. Digit. Signal Process..

[7] Mei S., Montanari A., Nguyen P.M. (2018). A mean field view of the landscape of two-layer neural networks. Proc. Natl. Acad. Sci. USA.

[8] Fort S., Hu H., Lakshminarayanan B. (2019). Deep ensembles: A loss landscape perspective. arXiv.

[9] Koch A.d.M., Koch E.d.M., Koch R.d.M. (2020). Why Unsupervised Deep Networks Generalize. arXiv.

[10] Poggio T., Kawaguchi K., Liao Q., Miranda B., Rosasco L., Boix X., Hidary J., Mhaskar H. (2017). Theory of deep learning III: Explaining the non-overfitting puzzle. arXiv.

[11] Allen-Zhu Z., Li Y., Song Z. A convergence theory for deep learning via over-parameterization. Proceedings of the International Conference on Machine Learning, PMLR.

[12] Zhou Z.H. (2021). Why over-parameterization of deep neural networks does not overfit?. Sci. China Inf. Sci..

[13] Franklin J. (2005). The elements of statistical learning: Data mining, inference and prediction. Math. Intell..

[14] Krizhevsky A., Sutskever I., Hinton G.E. (2012). Imagenet classification with deep convolutional neural networks. Adv. Neural Inf. Process. Syst..

[15] Huang Y., Cheng Y., Bapna A., Firat O., Chen D., Chen M., Lee H., Ngiam J., Le Q.V., Wu Y. (2019). Gpipe: Efficient training of giant neural networks using pipeline parallelism. Adv. Neural Inf. Process. Syst..

[16] Szegedy C., Liu W., Jia Y., Sermanet P., Reed S., Anguelov D., Erhan D., Vanhoucke V., Rabinovich A. Going deeper with convolutions. Proceedings of the IEEE Conference on Computer Vision and Pattern Recognition.

[17] Radford A., Wu J., Child R., Luan D., Amodei D., Sutskever I. (2019). Language models are unsupervised multitask learners. OpenAI Blog.

[18] Opper M. (1995). Statistical mechanics of learning: Generalization. The Handbook of Brain Theory and Neural Networks.

[19] Opper M. (2001). Learning to generalize. Front. Life.

[20] Advani M.S., Saxe A.M., Sompolinsky H. (2020). High-dimensional dynamics of generalization error in neural networks. Neural Netw..

[21] Spigler S., Geiger M., d’Ascoli S., Sagun L., Biroli G., Wyart M. (2018). A jamming transition from under-to over-parametrization affects loss landscape and generalization. arXiv.

[22] Geiger M., Spigler S., d’Ascoli S., Sagun L., Baity-Jesi M., Biroli G., Wyart M. (2019). Jamming transition as a paradigm to understand the loss landscape of deep neural networks. Phys. Rev. E.

[23] Liao Q., Poggio T. (2017). Theory II: Landscape of the empirical risk in deep learning. arXiv.

[24] Li H., Xu Z., Taylor G., Studer C., Goldstein T. (2018). Visualizing the loss landscape of neural nets. Advances in Neural Information Processing Systems.

[25] Blundell C., Cornebise J., Kavukcuoglu K., Wierstra D. Weight uncertainty in neural network. Proceedings of the International Conference on Machine Learning, PMLR.

[26] Gal Y., Ghahramani Z. Dropout as a Bayesian Approximation: Representing Model Uncertainty in Deep Learning. Proceedings of the 33rd International Conference on Machine Learning.

[27] Chollet F. (2017). Deep Learning with Python.

[28] Fukunaga K., Krishnaiah P.R., Kanal L.N. (1982). 15 Intrinsic dimensionality extraction. Handbook of Statistics.

[29] Li C., Farkhoor H., Liu R., Yosinski J. (2018). Measuring the intrinsic dimension of objective landscapes. arXiv.

[30] Facco E., d’Errico M., Rodriguez A., Laio A. (2017). Estimating the intrinsic dimension of datasets by a minimal neighborhood information. Sci. Rep..

[31] Levina E., Bickel P.J. Maximum likelihood estimation of intrinsic dimension. Proceedings of the Advances in Neural Information Processing Systems.

[32] Lombardi G., Rozza A., Ceruti C., Casiraghi E., Campadelli P. (2011). Minimum neighbor distance estimators of intrinsic dimension. Proceedings of the Joint European Conference on Machine Learning and Knowledge Discovery in Databases.

[33] Ceruti C., Bassis S., Rozza A., Lombardi G., Casiraghi E., Campadelli P. (2014). Danco: An intrinsic dimensionality estimator exploiting angle and norm concentration. Pattern Recognit..

[34] Ravichandran K., Jain A., Rakhlin A. (2019). Using Effective Dimension to Analyze Feature Transformations in Deep Neural Networks. https://openreview.net/pdf?id=HJGsj13qTE.

[35] Ansuini A., Laio A., Macke J.H., Zoccolan D., Wallach H., Larochelle H., Beygelzimer A., d’Alché-Buc F., Fox E., Garnett R. (2019). Intrinsic dimension of data representations in deep neural networks. Proceedings of the Advances in Neural Information Processing Systems.

[36] Ma X., Wang Y., Houle M.E., Zhou S., Erfani S., Xia S., Wijewickrema S., Bailey J. Dimensionality-driven learning with noisy labels. Proceedings of the International Conference on Machine Learning, PMLR.

[37] Baldassi L., Malatesta P., Zecchina (2021). Unveiling the Structure of Wide Flat Minima in Neural Networks. Phys. Rev. Lett..

[38] Altarabichi M.G., Nowaczyk S., Pashami S., Sheikholharam Mashhadi P., Handl J. (2024). Rolling the dice for better deep learning performance: A study of randomness techniques in deep neural networks. Inf. Sci..

[39] Zhuang D., Zhang X., Song S., Hooker S. (2022). Randomness in neural network training: Characterizing the impact of tooling. Proc. Mach. Learn. Syst..

[40] Géron A. (2019). Hands-on Machine Learning with Scikit-Learn, Keras, and TensorFlow: Concepts, Tools, and Techniques to Build Intelligent Systems.

[41] Ramachandran P., Zoph B., Le Q.V. (2017). Searching for activation functions. arXiv.

[42] Glorot X., Bordes A., Bengio Y. Deep sparse rectifier neural networks. Proceedings of the Fourteenth International Conference on Artificial Intelligence and Statistics.

[43] Masters D., Luschi C. (2018). Revisiting small batch training for deep neural networks. arXiv.

[44] Campadelli P., Casiraghi E., Ceruti C., Rozza A. (2015). Intrinsic dimension estimation: Relevant techniques and a benchmark framework. Math. Probl. Eng..

[45] Jolliffe I. (2005). Principal component analysis. Encycl. Stat. Behav. Sci..

[46] Cox M.A., Cox T.F. (2008). Multidimensional scaling. Handbook of Data Visualization.

[47] Tribello G.A., Ceriotti M., Parrinello M. (2012). Using sketch-map coordinates to analyze and bias molecular dynamics simulations. Proc. Natl. Acad. Sci. USA.

[48] Grassberger P., Procaccia I. (1983). Characterization of strange attractors. Phys. Rev. Lett..

[49] Fisher N.I. (1995). Statistical Analysis of Circular Data.

[50] Ciregan D., Meier U., Schmidhuber J. Multi-column deep neural networks for image classification. Proceedings of the 2012 IEEE Conference on Computer Vision and Pattern Recognition.

[51] Lee S., Purushwalkam S., Cogswell M., Crandall D., Batra D. (2015). Why M heads are better than one: Training a diverse ensemble of deep networks. arXiv.

[52] Ganaie M., Hu M., Malik A.K., Tanveer M., Suganthan P.N. (2021). Ensemble deep learning: A review. arXiv.

[53] Lakshminarayanan B., Pritzel A., Blundell C. (2016). Simple and scalable predictive uncertainty estimation using deep ensembles. arXiv.

[54] Wolpert D.H. (1992). Stacked generalization. Neural Netw..

[55] Salehi M., Razmara J., Lotfi S. (2020). A novel data mining on breast cancer survivability using MLP ensemble learners. Comput. J..

[56] Garipov T., Izmailov P., Podoprikhin D., Vetrov D.P., Wilson A.G., Bengio S., Wallach H., Larochelle H., Grauman K., Cesa-Bianchi N., Garnett R. (2018). Loss Surfaces, Mode Connectivity, and Fast Ensembling of DNNs. Proceedings of the Advances in Neural Information Processing Systems.

[57] Draxler F., Veschgini K., Salmhofer M., Hamprecht F. Essentially No Barriers in Neural Network Energy Landscape. Proceedings of the 35th International Conference on Machine Learning, PMLR.

[58] Guerra F.T. (2025). tgfrancesco/NN_intrinsic_dimension: V1.0.0. https://zenodo.org/records/15090926.

[59] Denti F., Doimo D., Laio A., Mira A. (2021). Distributional results for model-based intrinsic dimension estimators. arXiv.

